# Effects of the Number of Seeds per Berry on Fruit Growth Characteristics, Especially on the Duration of Stage II in Blueberry

**DOI:** 10.3390/plants7040096

**Published:** 2018-11-03

**Authors:** Kenichi Doi, Ryouichi Nozaki, Kouji Takahashi, Naoto Iwasaki

**Affiliations:** 1 Graduate School of Agriculture, Meiji University, Kawasaki, Kanagawa 213-8571, Japan; dken1.ten0115@gmail.com (K.D.); zi50zi100@yahoo.co.jp (R.N.); 2 School of Agriculture, Meiji University, Kawasaki, Kanagawa 213-8571, Japan; tanchon-runner@outlook.jp

**Keywords:** self-pollination, cross-pollination, pollen source, fruit weight, fruit ripening

## Abstract

In present research, differences in the number of seeds per berry (NSB), berry fresh weight (BW), days to ripening from flowering (DRF), and the duration of a slow growth phase (DS II) among pollen sources were investigated in highbush blueberry (*Vaccinium corymbosum*). NSB, as well as BW and DRF, were significantly different among the pollen sources. Analysis of covariance (ANCOVA) with NSB as the covariate showed significant interaction between the NSB and pollen sources on BW and DRF when self-pollination was included. However, ANCOVA without self-pollination showed no significant effect of the pollen source on BW and DRF. On the other hand, DS II was negatively correlated with NSB, and no significant interaction between NSB and pollen sources was found, even though self-pollination was included. Although the relationship between NSB and DS II appeared not to be statistically influenced by the different pollen sources, there seemed to be some difference between self- and cross-pollination. DS II shortened as the NSB increased, which may have led to a decrease in DRF.

## 1. Introduction

Seeds present inside fruits play crucial roles in fruit growth, maturation, and quality. In blueberry, individual berries exhibit a double sigmoid growth pattern similar to that in stone fruits, and their growth is similarly divided into three stages. Stage I is a rapid growth phase that begins soon after fruit set, followed by stage II, a slow growth phase (called the stone-hardening stage for stone fruits). In stage III, the berry grows rapidly again toward maturity. Since the embryo becomes germinable in stage II, it is believed that photosynthetic assimilates are used for the development of the seed, including the stone shell, such that pericarp enlargement is suppressed. In Japanese persimmon, whose fruits exhibit a double sigmoid growth pattern, stage II is also found in seedless cultivars [1,2]. In ‘Fuyu’, which is a seeded variety of the Japanese persimmon, no difference was noted in the fruit growth patterns caused by the difference in the number of seeds per fruit, although the dry matter content of the fruit (especially seeds) increased during stage II [3]. In kiwi fruit, treatment with N-(2-Chloro-4-pyridyl)-N’-phenylurea (CPPU), a synthetic indol-3-acetic acid (IAA), at day 20 after flowering resulted in continual fruit growth during stage II [4]. Therefore, there seems to be an involvement of factors other than seeds in stage II. Furthermore, the duration of stage II (DS II) is reportedly longer in the late-maturing varieties than in the early-maturing varieties of peach and blueberry [5,6]. The physiological factors involved in the suppression of pericarp enlargement in stage II remain to be fully elucidated. 

Blueberries contain many (about 50) small seeds per berry and they do not have a stone. Numerous reports have stated that the duration of the ripening period is affected by the number of seeds per berry (NSB) [7,8], while several reports state that ripening tends to occur at an earlier date in berries from earlier blooming flowers than in those from later blooming flowers within the same flower cluster in blueberry [9,10]. We have also previously reported that NSB and the percentage of viable seeds is higher in fruits produced by cross-pollination than in those produced by self-pollination or open-pollination, and the number of days to ripening from flowering (DRF) tends to be lower in cross-pollinated than in self-pollinated berries [11]. Therefore, berry growth in blueberry is apparently affected by the pollen source. Shirley et al. [12] reported that pollination by pollen from large fruiting cultivars produced larger fruit in ‘Hortblue Petite’ and that was evidence of metaxenia in blueberry. Metaxenia, which might be included in xenia because the definitions of xenia and metaxenia are somewhat different among researchers, is a phenomenon by which characteristics of the pollen parent are expressed in maternal tissues outside the embryo and endosperm in general [13]. It has been reported for cherry, pear, raspberry, and also for blueberry [14,15,16]. However, the details of the mechanism are not well known, and the phenomenon itself is doubtful in some cases.

The objective of this study was to determine the effects of the pollen source on the number of seeds formed and berry growth, especially DS II, and discuss the probability of metaxenia in highbush blueberry.

## 2. Results

### 2.1. Effects of Different Pollen Sources on Berry Characteristics

NSB, DRF, berry fresh weight (BW), and Brix significantly differed among seed parents depending on the pollen source (Table 1). NSB was the highest in ‘Bluejay’ pollinated by ‘Herbart’ and the lowest with open-pollination or self-pollination (Bluejay × Bluejay). BW and DRF were cultivar-specific, but they seemed to be influenced by NSB. With the exception of ‘Collins’, DRF tended to decrease with increased NSB. On the contrary, BW tended to increase with increased NSB. Brix varied among the pollen sources in ‘Darrow’, but not in Collins and Bluejay. Rind color, as represented by the b* value, did not vary among the pollen sources for all cultivars used (data not shown). In 2015, NSB, DRF and BW also significantly varied depending on the pollen source.

The result of ANCOVA with NSB as the covariate indicated the significant interaction between the pollen source and NSB in Bluejay, which means that the slope of the regression line between NSB and BW varied depending on the pollen sources. Therefore, further analysis of the effect of pollen sources on BW could not be advanced (Table 2). The analysis of the effect of the pollen source on BW was advanced in ‘Collins’ and ‘Darrow’, but BW adjusted by NSB did not differ significantly among pollen sources in both cultivars (Table 3).

The ANCOVA with NSB as the covariate in 2015 indicated different results for samples with and without self-pollination (Table 4). The interaction between the pollen source and NSB was significant when self-pollination was included, while it was not significant when self-pollination was excluded. However, the BW adjusted with NSB did not differ significantly among the pollen sources when self-pollination was excluded (Table 5). Similar ANCOVA results were obtained when analyzing the influence of pollen sources on DRF in 2013 and 2015 (data not shown).

### 2.2. Effect of NSB on Berry Growth

**Figure 1:**
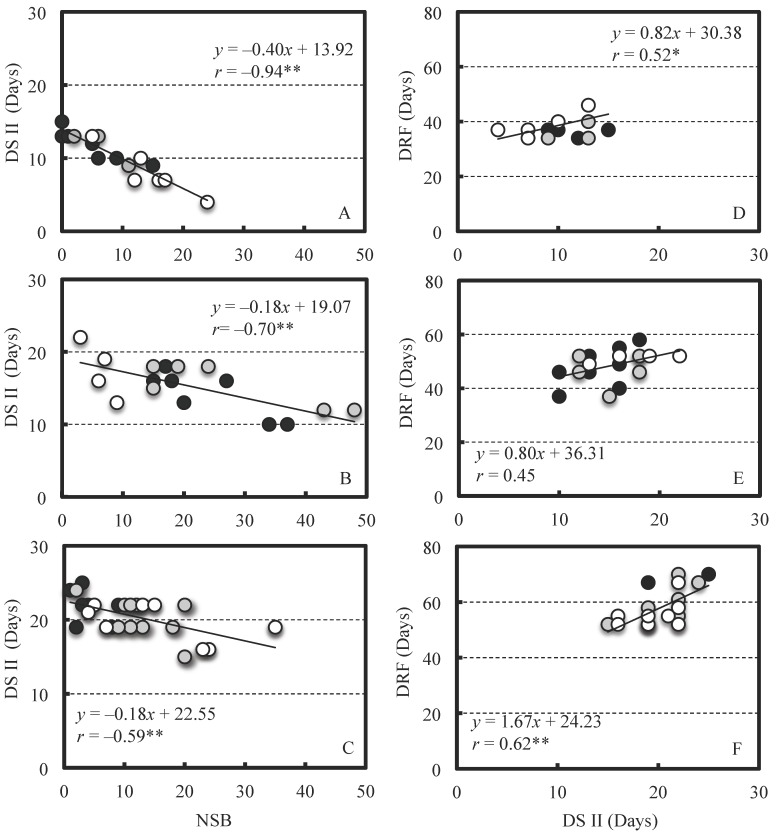
Relationship between the NSB and the DS II (**A**–**C**), the DS II and the DRF (**D**–**F**) in Collins (**A**,**D**), Bluejay (**B**,**E**) and Darrow (**C**,**F**) fruits. Fruits were produced by open-pollination (closed circles), hand-pollination with Herbert (shaded circles), and Bluejay (open circles) pollen. Correlation coefficient (*r*) with * or ** is significant at *p* = 0.05 or *p* = 0.01, respectively.

DS II tended to become shorter as the NSB increased in all cultivars used (Figure 1). Further, DS II appeared to be shorter in the early-maturing cultivar Collins than in the late-maturing cultivar Darrow, because the intercept of the regression line was lower. In addition, the slope of the regression line was steeper in the early-maturing cultivar Collins than in the other two cultivars. Since DRF showed a positive correlation with DSII, it appeared to decrease as NSB increased.

**Figure 2:**
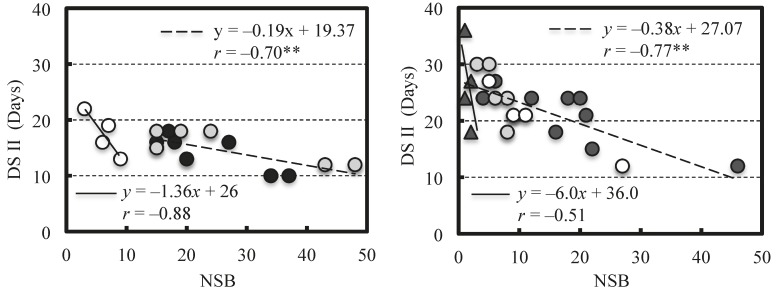
Difference in the effect of NSB on DS II between self-pollination and the other forms of pollination in Bluejay (**left**) and Herbert (**right**) as the seed parent. Each symbol in Bluejay represents the same as in Figure 1. In Herbert, each symbol indicates the following; open-pollination (open-circles); hand-pollination with Herbert (closed triangle), Bluejay (closed circles) and Darrow (shaded circles). ** indicates significant at *p* = 0.01.

The results of ANCOVA with NSB as a covariate showed that the interaction between the pollen source and NSB was not significant in all cultivars (Table 6). Therefore, no differences were noted in the slope of the regression lines among pollen sources and they were considered to be parallel. On the contrary, the regression line between the NSB and the DS II was significant in each cultivar (Collins; *p* = 1.6 × 10^−6^, Bluejay; *p* = 0.0021, Darrow; *p* = 0.0135); however, the effect of pollen source on the DS II adjusted by the covariate was not significant in all cultivars (Table 7). Similar ANCOVA results were obtained in 2015, and no significant interaction between pollen source and NSB detected (Table 8). There were no significant differences in adjusted DS II by NSB among pollen sources (*p* = 0.7280). Therefore, a difference in the pollen source did not affect the relationship between DS II and NSB. In Bluejay, the relationship between NSB and DS II seemed to be different between self-pollination and the other forms of pollination (Figure 2). However, the correlation coefficient was not significant for self-pollination due to the limited data. The same trend was found in 2015 (Herbert), and the range of NSB variation in self-pollination was more limited than that of Bluejay. 

## 3. Discussion

The presence of seeds inside fruits has been considered to affect fruit growth and quality [7,8]. However, it is difficult to explain the quantitative relationship between the seeds and the size or quality of the fruit, even in a given cultivar. The results of this study indicate that a difference in NSB induced by pollination from different sources of pollen results in differences in BW and DRF. Then, we employed analysis of covariance (ANCOVA) with NSB as a covariate to clarify the influence of the pollen source on the relationship between NSB and the traits and fruit quality characteristics. The ANCOVA results with self- and cross-pollination included indicated a significant interaction between the pollen source and NSB, which suggests that the other factors also affect the BW and DRF. Therefore, the influence of NSB on the fruit quality characteristics may be different between self- and cross-pollination. Further research is necessary to elucidate the effects of the seeds on the fruit quality characteristics.

Tukey [5] reported that the DS II varied among cultivars of peach and was longer with late fruit maturity. In this study, since similar results were obtained, it was believed that the DS II would also be longer in the late-maturing cultivars in blueberry. However, negative correlations between NSB and DS II were found in the four cultivars used in this study; as more seeds are present in the berry, stage II becomes shorter, and this is considered to result in earlier ripening. Tukey [17] also reported that the destruction of the embryo early in stage II resulted in fruit abscission, but when this occurred between stages II and III it induced earlier fruit ripening. Therefore, the presence of the seed was considered to result in a longer DS II in peach. The IAA concentrations reportedly increase with seed enlargement during stage II in ‘Berkeley’ [18], which may accelerate berry growth. Therefore, it seems that there is some relationship between the seed presence and DS II. However, the competition for photosynthates reportedly occurs between seedy and seedless fruits from around the beginning of stage II when both fruits are on the same branch [19]. The effects induced by the seeds on stage II vary among fruit species, so there may be several factors at work. On the contrary, no difference was found among the pollen sources in the relationship between NSB and DS II, and one regression line could be adapted by combining all the data in each cultivar used as a seed parent by ANCOVA. This result indicated that a difference in pollen source did not affect the relationship between DS II and NSB, even if self- and cross-pollination were included. However, the difference between self- and the other forms of pollinations in the relationship between DS II and NSB was not detected by ANCOVA in the present study, there seemed appeared to be some difference between them as far as seeing the Figure 2. 

If the difference between self- and cross-pollination is assumed as the difference of pollen source, there may be metaxenia. However, as far as the results of cross-pollination, there is no evidence of metaxenia in highbush blueberry. In conclusion, the pollen source apparently affects NSB, and which is considered to result in the differences in BW and DRF. In the present study, the number of fruits used was limited, especially with regard to self-pollination, because self-pollination was difficult to produce fruit in highbush blueberry. Further experiments will be necessary to elucidate details.

## 4. Materials and Methods

### 4.1. Plant Material

Experiments were conducted in 2013 and 2015 using the highbush blueberry (*Vaccinium corymbosum*) varieties Collins, Bluejay, Darrow and Herbert as seed parents. Self- or cross-pollination was conducted in experimental fields at Meiji University. The age of the Bluejay trees was 10 years, while that of the Collins, Darrow, and Herbert trees was 15 years. All trees were potted in 45 L pots using peat moss and received similar cultural treatment including nutrition and plant protection. 

### 4.2. Pollination Treatments and Pollen Sources

In 2013, Collins, Bluejay and Darrow as seed parents were hand-pollinated with pollen from Bluejay and Herbert, as well as subjected to open-pollination in early April. In 2015, Herbert was used as the seed parent, while Herbert, Bluejay and Darrow were used as pollen sources for hand-pollination. The flowers of the seed parent were emasculated on the day before flowering, and then covered with a small bag made of paraffin paper. Hand-pollination was performed on the day following emasculation, and a small paper bag was attached to the flower cluster again for approximately one week to prevent further pollination. Pollination was performed by lightly touching the stigmatic surface several times with a small brush bearing the pollen. Twenty-five clusters, approximately 100 florets, were used for each pollen source.

One tree each of Herbert, Bluejay and Darrow was transferred to a greenhouse in early March to accelerate flower bud development, and anthers were collected from the flowers just before flowering. The anthers were opened in an incubator maintained at 25 °C for 24 h and the dried pollen was stored in a freezer at −70 °C or −36 °C until use. The stored pollen was transferred to a refrigerator the evening before the day of pollination and returned to room temperature the next morning. The germination rate of the pollen was tested on agar medium containing 1% agar, 0.01% of H_3_BO_3_, and 20% of sucrose. The pollen germination rate was 21.3% and 39.8% in Herbert and Bluejay, respectively, and was significantly different (*p* = 0.05) between varieties in 2013. However, in 2015 the percentage of fruit set was 88.6, 74.0, and 65.4% in cross-pollination by Bluejay, Darrow, and open-pollination, respectively, and there was no significant difference among the forms of pollination. In addition, there seemed to be no effect of bagging on the fruit set, because cross-pollination was followed by bagging, but open-pollination was not.

### 4.3. Measurements

**Figure 3:**
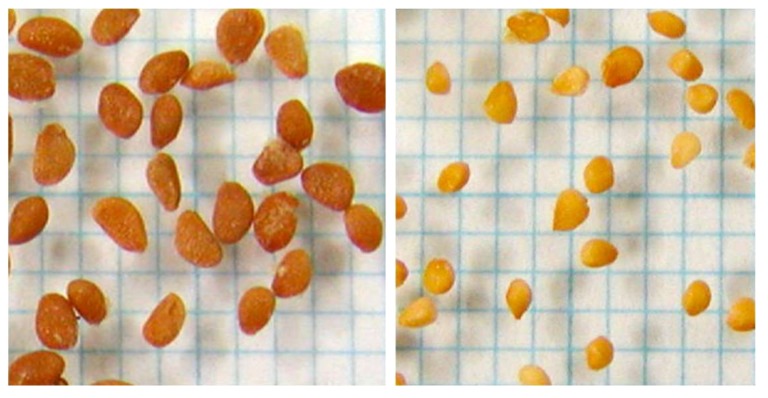
Difference in seed coat color between brownish (**left**) and whitish (**right**) seeds.

All the berries were harvested at the stage when they were fully colored to the base of the peduncle and DRF was then calculated. Berry fresh weight (BW), rind color by CIELAB (L*, a*, b*), Brix, and NSB were determined for all harvested berries. Seeds were collected from every berry and dried at room temperature for about one month. All the seeds were screened through a 26 mesh sieve. Seeds remaining on the sieve were classified as large and those that passed through were classified as small. The large seeds were classified as either brownish or whitish by seed coat color (Figure 3).

The transverse diameter of 10 randomly selected berries was measured using vernier calipers every three days from immediately after fruit set through to harvest to determine the DS II. Some of the fruits were dropped before ripening and were eliminated from data analysis. Any three-day period in which the difference in diameter was less than 3% was defined as non-enlargement, and DS II was taken as the period of non-enlargement. 

### 4.4. Data Analysis

All data analyses were performed using the BellCurve for Excel software (Social Survey Research Information Co., Ltd., Tokyo, Japan). Mean separations were performed using the Tukey–Kramer test when analysis of variance (ANOVA) was significant at *p* = 0.05 or *p* = 0.01. The effect of the differences in pollen source on BW, DFR and DS II was analyzed by analysis of covariance (ANCOVA) with NSB as the covariate because NSB also differs depending on the pollen source. When analyzing the influence of the number of seeds on the BW and DRF by ANCOVA, the number of seeds was converted into a natural logarithm, because they showed a logarithmic relationship.

## Figures and Tables

**Table 1:** Differences in the number of brownish seeds per berry (NSB), days to ripening from flowering (DRF), and fruit quality characteristics among pollinated pollen sources in the experiments of 2013 and 2015.

Seed Parent	Pollen Source	Number of Brownish Seeds (no.)	Days to Ripening from Flowering	Berry Weight (g)	Brix (%)
2013					
Collins	Open (27) ^z^	5.3 ± 0.83 b ^y^	55.2 ± 0.85 a	1.2 ± 0.05 b	13.5 ± 0.23 a
	Bluejay (43)	19.2 ± 1.99 a	54.5 ± 0.68 a	2.1 ± 0.07 a	13.8 ± 0.18 a
	Herbert (45)	13.8 ± 0.19 b	55.9 ± 0.69 a	1.8 ± 0.07 b	13.8 ± 0.19 a
Bluejay	Open (51)	12.1 ± 1.79 ab	60.6 ± 0.87 ab	1.2 ± 0.05 b	12.6 ± 0.18 a
	Bluejay (13)	8.0 ± 1.21 b	64.7 ± 2.02 a	1.1 ± 0.08 b	12.4 ± 0.30 a
	Herbert (22)	21.2 ± 4.97 a	58.0 ± 1.09 b	1.5 ± 0.13 a	12.4 ± 0.44 a
Darrow	Open (26)	5.7 ± 1.37 b	75.5 ± 1.22 a	1.9 ± 0.12 b	10.6 ± 0.27 b
	Bluejay (57)	13.4 ± 1.21 a	71.2 ± 0.67 b	2.3 ± 0.08 a	11.4 ± 0.13 a
	Herbert (41)	10.0 ± 1.18 ab	73.3 ± 0.99 ab	2.1 ± 0.09 ab	11.1 ± 0.13 ab
2015					
Herbert	Herbert (43)	2.2 ± 0.18 c	68.5 ± 0.85 a	0.94 ± 0.04 c	-
	Bluejay (75)	27.4 ± 2.26 a	59.3 ± 0.67 b	1.39 ± 0.05 a	-
	Darrow (81)	13.6 ± 1.03 b	61.4 ± 0.77 b	1.21 ± 0.05 b	-
	Open (87)	7.8 ± 0.63 c	60.3 ± 0.61 b	0.98 ± 0.07 c	-

^z^ Numbers in parentheses indicate the number of berries used for analysis. ^y^ Different letters within the column and seed parent indicate significant difference at *p* = 0.05 by the Tukey–Kramer test.

**Table 2:** Results of AVCOVA with NSB as a covariate determining the parallelism of the regression lines between NSB and BW in different pollen sources (2013 experiment).

	df	Mean Square	F-value	Probability
Collins				
Pollen source: A	2	0.0690	0.6041	0.5483
Ln (NSB): B	1	10.0185	87.7344	6.3 × 10 ^−16^ **
A × B	2	0.0289	0.2528	0.7770
Residuals	118	0.1142		
Bluejay				
Pollen source: A	2	0.2557	4.1672	0.0191
Ln (NSB): B	1	2.4738	40.3156	1.4 × 10 ^−8^ **
A × B	2	0.5424	8.8400	0.0004 **
Residuals	80	0.0614		
Darrow				
Pollen source: A	2	0.1823	1.0165	0.3650
Ln (NSB): B	1	20.8407	116.2287	2.8 × 10 ^−19^ **
A × B	2	0.1094	0.6104	0.5448
Residuals	118	0.1793		

** indicates significant at *p* = 0.01.

**Table 3:** Results of ANCOVA with NSB as a covariate determining the differences in adjusted BW among the pollination by different sources of pollen (2013 experiment).

	df	Mean Square	F-value	Probability
Collins				
Pollen source	2	0.0541	0.4797	0.6201
Ln (NSB)	1	11.9638	106.0910	3.3 × 10 ^−18^ **
Residuals	120	0.1128		
Darrow				
Pollen source	2	3.3654	0.1652	0.8479
Ln (NSB)	1	1647.0494	80.8417	4.3 × 10 ^−15^ **
Residuals	120	20.3738		

** indicate significant at *p* = 0.01.

**Table 4:** Results of ANCOVA determining the differences in the regression coefficients between NSB and BW among pollen sources in the 2015 experiment with or without self-pollination.

	df	Mean Square	F-value	Probability
With self-pollination				
Pollen source: A	3	0.7520	8.7712	0.00001 **
Ln (NSB): B	1	8.4055	98.0405	5.9 × 10 ^−20^ **
A × B	3	0.2666	3.1099	0.0269 *
Residuals	274	0.0857		
Without self-pollination				
Pollen source: A	2	0.0611	0.7011	0.4971
Ln (NSB): B	1	17.5101	200.8371	2.7 × 10 ^−33^ **
A × B	2	0.1194	1.3691	0.2564
Residuals	233	0.0872		

* and ** indicate significant at *p* = 0.05 and *p* = 0.01 respectively.

**Table 5:** Results of ANCOVA determining the differences in the adjusted BW among the pollen sources in the 2015 experiment without self-pollination.

	df	Mean Square	F-value	Probability
Pollen source	2	0.0396	0.4527	0.6365
Ln (NSB)	1	17.4503	199.5241	3.3 × 10 ^−33^ **
Residuals	235	0.0875		

** indicates significant at *p* = 0.01.

**Table 6:** Results of AVCOVA with NSB as the covariate determining the parallelism of the regression lines between NSB and DSII in each pollination by different sources of pollen.

	df	Mean Square	F-value	Probability
Collins				
Pollen source (A)	2	0.6444	0.5031	0.6160
NSB (B)	1	45.1936	35.2829	4.9 ×10^−5^ **
A × B	2	0.8747	0.6829	0.5224
Residuals	13	1.2809		
Bluejay				
Pollen source (A)	2	5.5544	1.4549	0.2718
NSB (B)	1	58.6199	15.3541	0.0020 **
A × B	2	14.5041	3.7990	0.0527
Residuals	12	3.8179		
Darrow				
Pollen source (A)	2	1.8761	0.3661	0.6982
NSB (B)	1	19.7509	3.8545	0.0644
A × B	2	2.1233	0.4144	0.6666
Residuals	19	5.1241		

** indicates significant at *p* = 0.01.

**Table 7:** Results of ANCOVA with NSB as a covariate determining the differences in adjusted DSII in each pollination by different sources of pollen.

	df	Mean Square	F-value	Probability
Collins				
Pollen source	2	0.1695	0.1382	0.8720
NSB	1	71.1657	58.0123	1.6 ×10^−6^ **
Residuals	15	1.2267		
Bluejay				
Pollen source	2	9.9801	1.8674	0.1910
NSB	1	75.6774	14.1599	0.0021 **
Residuals	14	5.3445		
Darrow				
Pollen source	2	0.0754	0.0156	0.9846
NSB	1	35.2276	7.2810	0.0135 *
Residuals	21	4.8383		

* and ** indicate significant at *p* = 0.05 and *p* = 0.01, respectively.

**Table 8:** Results of ANCOVA determining the parallelism of the regression lines between NSB and DS II among pollen sources in the 2015 experiment.

	df	Mean square	F-value	Probability
Pollen source: A	3	20.0138		0.2810
NSB: B	1	87.8476	6.1172	0.0250 *
A × B	3	36.0401	2.5096	0.0957
Residuals	16	14.3606		

* indicates significant at *p* = 0.05.
